# A Randomized Trial to Assess the Impact of a Package of Diagnostic Tools and Diagnostic Algorithm on Antibiotic Prescriptions for the Management of Febrile Illnesses Among Children and Adolescents in Primary Health Facilities in Burkina Faso

**DOI:** 10.1093/cid/ciad331

**Published:** 2023-07-25

**Authors:** Francois Kiemde, Daniel Valia, Berenger Kabore, Toussaint Rouamba, Alima Nadine Kone, Seydou Sawadogo, Adelaide Compaore, Olawale Salami, Philip Horgan, Catrin E Moore, Sabine Dittrich, Juvenal Nkeramahame, Piero Olliaro, Halidou Tinto

**Affiliations:** Clinical Research Unit of Nanoro, Institut de Recherche en Sciences de la Santé, Nanoro, Burkina Faso; Clinical Research Unit of Nanoro, Institut de Recherche en Sciences de la Santé, Nanoro, Burkina Faso; Clinical Research Unit of Nanoro, Institut de Recherche en Sciences de la Santé, Nanoro, Burkina Faso; Clinical Research Unit of Nanoro, Institut de Recherche en Sciences de la Santé, Nanoro, Burkina Faso; Clinical Research Unit of Nanoro, Institut de Recherche en Sciences de la Santé, Nanoro, Burkina Faso; Clinical Research Unit of Nanoro, Institut de Recherche en Sciences de la Santé, Nanoro, Burkina Faso; Clinical Research Unit of Nanoro, Institut de Recherche en Sciences de la Santé, Nanoro, Burkina Faso; FIND, Geneva, Switzerland; FIND, Geneva, Switzerland; Nuffield Department of Medicine, Big Data Institute, University of Oxford, Oxford, United Kingdom; Evidence and Impact Oxford, Oxford, United Kingdom; Nuffield Department of Medicine, Big Data Institute, University of Oxford, Oxford, United Kingdom; Centre for Neonatal and Pediatric Infection, Institute for Infection and Immunity, St George's University of London, London, United Kingdom; FIND, Geneva, Switzerland; Centre for Tropical Medicine and Global Health, Nuffield Department of Medicine, University of Oxford, Oxford, United Kingdom; Deggendorf Institute of Technology, European Campus Rottal Inn, Pfarrkirchen, Germany; FIND, Geneva, Switzerland; FIND, Geneva, Switzerland; International Severe Acute Respiratory and Emerging Infection Consortium, Pandemic Sciences Institute, University of Oxford, Oxford, United Kingdom; Clinical Research Unit of Nanoro, Institut de Recherche en Sciences de la Santé, Nanoro, Burkina Faso

**Keywords:** antibiotic prescription, antibiotic reduction, antimicrobial resistance, acute febrile illness

## Abstract

**Background:**

Low- and middle-income countries face significant challenges in differentiating bacterial from viral causes of febrile illnesses, leading to inappropriate use of antibiotics. This trial aimed to evaluate the impact of an intervention package comprising diagnostic tests, a diagnostic algorithm, and a training-and-communication package on antibiotic prescriptions and clinical outcomes.

**Methods:**

Patients aged 6 months to 18 years with fever or history of fever within the past 7 days with no focus, or a suspected respiratory tract infection, arriving at 2 health facilities were randomized to either the intervention package or standard practice. The primary outcomes were the proportions of patients who recovered at day 7 (D7) and patients prescribed antibiotics at day 0.

**Results:**

Of 1718 patients randomized, 1681 (97.8%; intervention: 844; control: 837) completed follow-up: 99.5% recovered at D7 in the intervention arm versus 100% in standard practice (*P* = .135). Antibiotics were prescribed to 40.6% of patients in the intervention group versus 57.5% in the control arm (risk ratio: 29.3%; 95% CI: 21.8–36.0%; risk difference [RD]: −16.8%; 95% CI: −21.7% to −12.0%; *P* < .001), which translates to 1 additional antibiotic prescription saved every 6 (95% CI: 5–8) consultations. This reduction was significant regardless of test results for malaria, but was greater in patients without malaria (RD: −46.0%; −54.7% to −37.4%; *P* < .001), those with a respiratory diagnosis (RD: −38.2%; −43.8% to −32.6%; *P* < .001), and in children 6–59 months old (RD: −20.4%; −26.0% to −14.9%; *P* < .001). Except for the period July–September, the reduction was consistent across the other quarters (*P* < .001).

**Conclusions:**

The implementation of the package can reduce inappropriate antibiotic prescription without compromising clinical outcomes.

**Clinical Trials Registration:**

clinicaltrials.gov; NCT04081051.

## Teaser

In this paper, we report the primary findings of a randomized controlled trial to evaluate the impact of point-of-care tests combined with a training-and-communication package in reducing antibiotic prescriptions for fever management in Burkina Faso.

Fever is the most common presenting clinical symptom in children attending healthcare facilities in sub-Saharan Africa [1–3]. Without appropriate diagnostics in routine practice in peripheral healthcare settings to identify the causing pathogen (exception is malaria), patients are managed clinically and according to the national guidelines, based on World Health Organization (WHO) guidelines [4]. Limited diagnostic capacity for nonmalaria infections, compounded with fear of overlooking a potentially life-threatening and treatable bacterial infection, can lead to inappropriate use of antibiotics [5,6]. While antibiotics play an important role in the control of infections, their inappropriate use and overuse threatens their effectiveness [2,5–11], and is regarded as a global public health concern [12].

The introduction of rapid diagnostic tests (RDTs) for malaria into routine healthcare is a major innovation in the management of febrile diseases in malaria-endemic areas; their introduction has considerably reduced unnecessary prescription of antimalarials [13]. However, the “test-treat-and-track” strategy for malaria has created uncertainty around the diagnosis and management of “nonmalaria infections,” especially where the etiologies of febrile diseases are varied and remain unknown [14]. A study in Burkina Faso found that antibiotics are almost always prescribed to febrile children attending outpatient clinics, at odds with national guidelines for the management of febrile diseases [15], given the limitation of healthcare workers to correctly diagnose nonmalaria diseases. Given the acceleration of antimicrobial resistance (AMR) in low- and middle-income countries (LMICs) [16,17], there is a clear need to differentiate bacterial from nonbacterial causes of fever in order to better target antibiotic treatment and reduce AMR.

Capitalizing on the successful management of malaria and human immunodeficiency virus (HIV) due to the deployment of the respective RDTs [18,19], the introduction of point-of-care tests (POCTs) that can be implemented in rural areas (without laboratory facilities) to diagnose other causes (bacterial or viral) of fever will create an opportunity to improve the management of febrile diseases in areas without laboratory facilities. Correct and timely diagnosis of febrile diseases remains essential for appropriate therapeutic prescription within a reasonable time to avoid fatalities due to late and/or discordant prescription of antimicrobials.

This clinical study was conducted to assess the impact of a package of interventions, including a combination of diagnostic tests, algorithms, and the training-and-communication (T&C) package, on clinical outcomes and antibiotic prescription patterns in febrile patients attending healthcare clinics in 2 health facilities in Burkina Faso.

## METHODS

### Study Design

A prospective, comparative, open-label, 2-armed, randomized controlled diagnostic trial was conducted from 7 September 2020 to 20 September 2021 at the outpatient clinics of the medical center Saint Louis of Temnaore and the health facility of Pella in the health district of Nanoro in Burkina Faso. Briefly, any outpatient attending the participating health centers in the health district of Nanoro, fulfilling the inclusion criteria below, was invited to participate in the study by signing the informed consent form [20]. This trial has been registered at clinicaltrials.gov on 6 September 2019 under the following identifier: NCT04081051.

### Participant Eligibility Criteria

All children and adolescents from 6 months to less than 18 years attending the outpatient clinics of the 2 medical centers, with acute febrile disease defined as fever (current or within the past 7 d) with no focus or suspected respiratory tract infection (RTI), were eligible to be enrolled in the study. In addition, parents or guardians of participants consented and expressed their willingness to provide blood and other biological samples (dependent on current infections), to adhere to study procedures, and to return for follow-up visits at the recruitment facilities on day 7 (±2 d) [20]. For participants over 12 years, an assent form was signed by the participants themselves in addition to the consent form signed by the parents or guardians.

### Randomization and Assessment

Eligible consenting participants were randomized to receive either the intervention package or standard care in a 1:1 ratio in block sizes of 64, 96, or 128 participants. In order to respect the attendance figure at the recruitment sites (children <5 y represented 75% of consultation the previous year), participants were randomized by age group—that is, children under 5 years (1083 participants) and from 5 years to less than 18 years (635 participants). Briefly, individual randomization codes were generated by the FIND data-management team and sent to the Clinical Research Unit of Nanoro (CRUN) data-management team for printing and placing in envelopes. The data-management team at CRUN was not involved in the implementation of the study and had no contact with the clinical trial team. The allocation of randomization code was made in chronological order by allocating each subsequent number to the subsequent participant.

All participants in both arms received a prescription on day 0 based on clinical decision (supported by a POCT in the intervention arm) and were followed up on day 7 for evaluation of the clinical outcomes and adherence to prescriptions. Nevertheless, to assess adherence to antibiotic prescriptions in the intervention arm, procurement of antibiotics was collected for those who received a prescription and those without an antibiotic prescription at day 0. For clinical recovery at day 7, the following definition was used: being alive, with no fever, amendment of day 0 symptoms.

### Intervention

**Figure 1.**
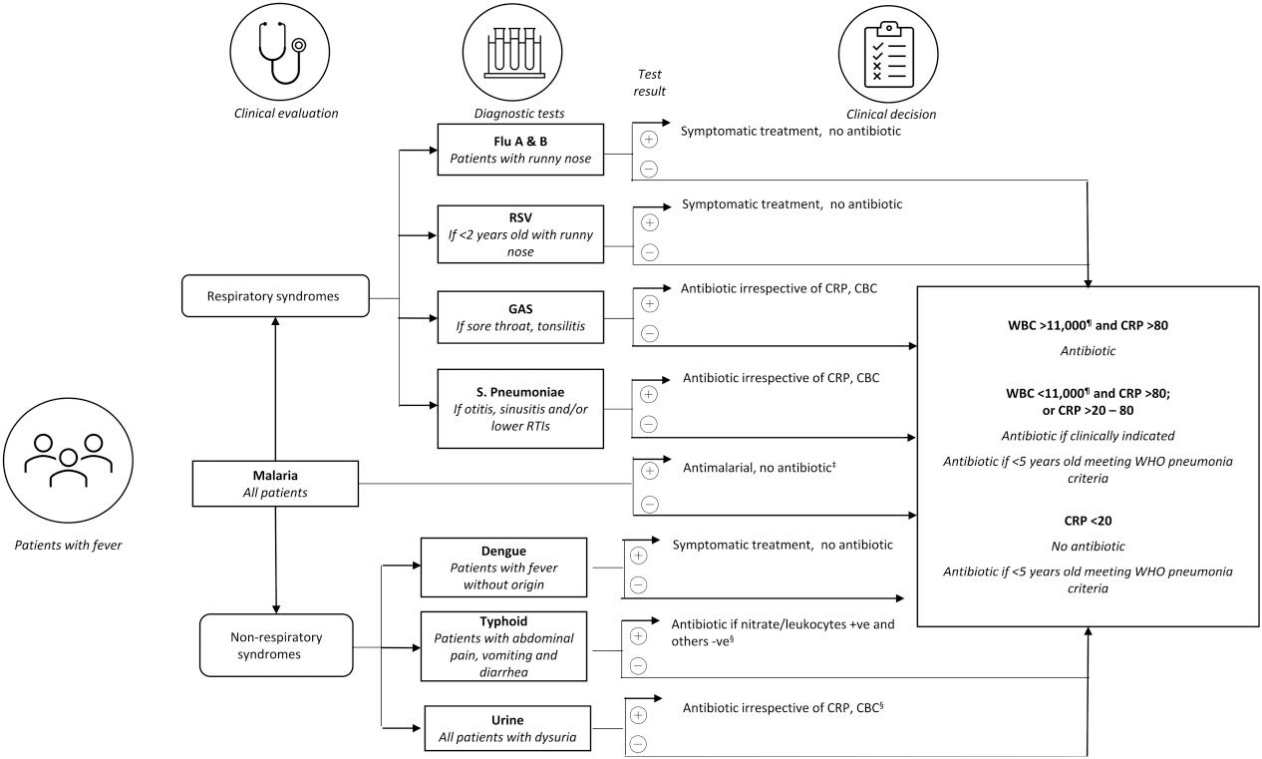
Diagnostic algorithm used in prescription decision making. Abbreviations: CBC, complete blood count; CRP, C-reactive protein (mg/L); GAS, group A streptococci; RSV, respiratory syncytial virus; RTI, respiratory tract infection; WBC, white blood cell count (per μL); WHO, World Health Organization.

**Figure 2.**
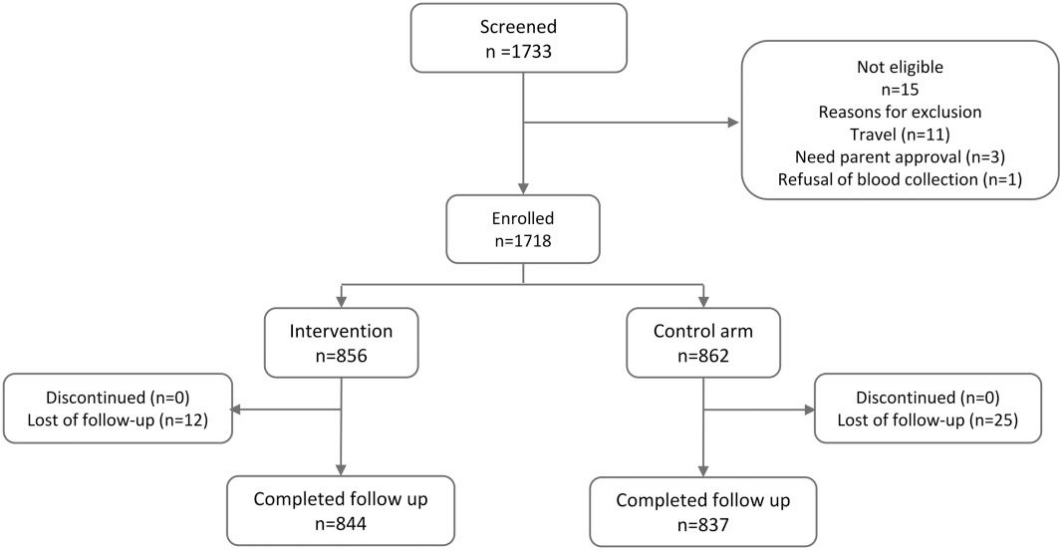
Participant disposition.

The trial intervention consisted of (1) diagnostic tests for common causes of fever in Burkina Faso (Figure 1), (2) diagnostic and clinical algorithms, and (3) a T&C package for healthcare workers and patients/caregivers [20]. The POCTs in the diagnostic algorithm (Figure 1) were selected based on local fever epidemiology, availability of the tests, and approval for use. The diagnostic algorithm (Figure 1) guided health workers on whether to prescribe an antibiotic or not depending on the results of malaria RDT and other pathogen-specific POCT. Non–pathogen-specific POCTs, such as white blood cell (WBC) total and differential count (WBC/diff) and C-reactive protein (CRP), were also used to guide antibiotic treatment when pathogen-specific POCTs were negative [20]. The use of pathogen-specific POCTs was based on the clinical presentation of the participant to support the clinical diagnostic, based on biological evidence. Non–pathogen-specific POCTs were used when the pathogen-specific tests did not advise the prescription of an antibiotic to not miss any potential bacterial infection. To optimize the flow of patients and minimize the time of patients’ assessment in the intervention arm, clinical examination and the conducting of POCTs were done by a team of trained staff. Prescriptions were completed by study nurses responsible for medical care in the intervention arm. To avoid a long waiting time in this arm, POCTs were performed by trained nurse-assistants and the management of participants by trained nurses. The requested times to perform each POCT were as follows: 5 minutes for WBC, 3 minutes for CRP testing, 2 minutes for urine dipstick testing, and a maximum of 15 minutes for other pathogen-specific POCTs. For children under 5 years of age, the diagnosis of pneumonia was based on the WHO clinical definition [4]: if met, an antibiotic (amoxicillin) was prescribed without further testing.

In the control arm, malaria RDT was the only diagnostic tool available for the diagnosis of fever episode as recommended by the National Malaria Control Program (PNLP) in standard practice. Except for malaria infection, the prescription of an antibiotic in the control arm was based on standard clinical practice based on clinical signs and symptoms. For children aged 6–59 months, the standard practice was the Integrated e-Diagnostic Approach (IeDA), which is based on Integrated Management of Childhood Illness (IMCI) guidance [21]. In this arm, nurses of the health facilities were responsible for the management of patients.

In both arms, adverse events (AEs) or serious AEs (SAEs) were defined as no improvement, worsening, death, or requirement of hospitalization of the participant within 7 days of follow-up. In case of AEs or SAEs, the participant was excluded to follow-up and managed by the study clinician based on the national guideline.

### Training-and-Communication Package

We assumed that support for the behaviors of patients and healthcare workers would be needed to understand and maximize the benefits of new diagnostics and algorithms. As such, we conducted a pre-intervention, qualitative research study to investigate the social, economic, and cultural factors that support or hinder patients’ adherence to prescriptions and the communication of adherence messages from healthcare workers, with additional qualitative components nested into the clinical trial. A T&C intervention package was then developed based on this research, which consisted of a set of communication messages presented to patients in local languages at the point of prescribing, and associated training for healthcare workers to deliver the messages. The outcomes of this qualitative work are also reported in this supplement issue (Compaoré et al).

Study nurses in the intervention arm were trained on the use of the algorithm and the intervention package designed by the social science team and on the design of message addressed to each participant, parent, or guardian, based on their knowledge of antibiotics and their understanding of the prescription made. At the day 7 visit, nurses collected information on antibiotic procurement in both arms and performed a pill count of remaining medications.

For the secondary objective of adherence to the prescription, adherence was defined as buying or obtaining the prescribed medicines and taking those medicines in line with the duration, frequency, and dosage stated in the prescription. For patients whose prescription did not include an antibiotic, this meant that, to adhere, they must have not subsequently bought/obtained an antibiotic. As part of the qualitative component of work, social scientists conducted in-depth interviews (IDIs) with study patients, through which they learned if the patient had adhered to the prescription. This information was added to the patient’s clinical record form for quantitative analysis of secondary objectives, and further qualitative analysis was conducted on a sample of interview recordings. In this paper, we limit reporting to the quantitative analysis.

Prescription adherence is calculated from the data collected primarily through the patient’s IDI or direct report to nurse; where that is not available, the pill count (>90%) is used and is calculated for patients who were prescribed an antibiotic, and not prescribed an antibiotic as described above. The results of this primary analysis and subcomponent analysis are provided in the discussion (adherence).

### Sample Size and Statistical Analysis

The sample size was calculated based on the expected relative reduction (risk difference [RD]) in antibiotic prescription and the precision of the estimation of the measured reduction, at a 5% significance level and 80% power [20]. Estimated antibiotic prescription practices used for sample size calculation were collected in outpatient clinic books at study sites. The formula used to determine the sample size has been previously published by Zhou et al [22].

Based on data collected in the field, the expected antibiotic prescription rate was 77% in the control group [20]. The sample size was calculated based on an estimate of the expected relative reduction in antibiotic prescriptions in the intervention arm of 30% *versus* the control arm. The sample size calculation allowed for 10% loss to follow-up, with a margin of precision of 5%, which is n = 859 *per* arm (ie, 1718 children and adolescents overall). Monthly attendance for fever reported the previous year was used to distribute the sample size for 1-year recruitment covering the range of different transmission seasons.

Data analysis was performed using R version 4.2.1 (R Foundation for Statistical Computing, Vienna, Austria). For the quantitative data, the descriptions were performed by using means or medians accordingly. Absolute values and percentages were used to describe qualitative data.

Primary outcome variables (clinical outcome after the follow-up visit by day 7 and antibiotic prescription; per protocol analyses) were compared between the intervention and routine arms in terms of both relative (risk ratio [RR]) and absolute (RD) effects, with 95% confidence intervals (CIs); numbers needed to test were calculated as 1/RD, with 95% CI. Fisher's exact test was used to compare proportions by estimating the *P* value.

### Ethics Statement

This study was approved by the National Ethical Committee for Health Research (Deliberation no. 2020-01-010) and the Institutional Ethical Committee for Research in Health Sciences (N/Ref. A09-2029/CEIRES). The study was also approved by the Oxford Tropical Research Ethical Committee (OxTREC reference: 52-19). The study was conducted in accordance with the following guidelines: (1) consensus ethical principles derived from international guidelines including the Declaration of Helsinki, (2) International Conference on Harmonization Good Clinical Practice guidelines (ICH GCP E6 [R2]), and (3) applicable laws and regulations. Written informed consent was obtained from all parents/guardians before enrollment into the study.

## RESULTS

### Baseline Demographics and Characteristics

From September 2020 to September 2021, 1733 participants were screened and 1718 enrolled. Of these, 856 (653 aged from 6 to 59 mo, and 203 from 5 y to <18 y) were randomized in the intervention arm and 862 (649 and 213, respectively) in the standard-practice arm (control arm). Fifteen participants were not enrolled for the reasons outlined in Figure 2. The per-protocol (PP) population included 1681 participants (97.8% of those randomized: 844 in the intervention arm and 837 in the routine-practice arm). Twice as many participants were lost to follow-up in the control arm compared with the intervention arm (25 and 12, respectively). Few patients needed hospitalization or returned to the recruitment site for being unwell (unscheduled visit); these numbers were lower in the intervention arm (hospitalization: 2; unscheduled visit: 11) than in the control arm (hospitalization: 6; unscheduled visit: 17), although this difference was not statistically significant (Figure 2).

Since testing was performed by nurse assistants based on clinical presentation and nurses were responsible for clinical assessment and management of participants, the time required for testing was 15–20 minutes. The waiting time of the participants at the health centers was 20–30 minutes compared to 10–15 minutes in the routine arm. Nevertheless, for children under 5 years with suspicion of pneumonia based on the WHO definition in the intervention arm, the waiting time was similar to that in the routine arm (10–14 min).

After fever (97.1%; 1668/1718), the most common reasons for the clinic consultation were sneezing and rhinorrhea (43.0%; 739/1718), cough (39.1%; 671/1718), abdominal pain (22.8%; 392/1718), and diarrhea (21.7%; 373/1718) (Table 1).

**Table 1.** Baseline Characteristics of the Study Population in the Intervention and Control Arms (Standard Routine)

Characteristics	Total, N (%)	Study Arm, n/N (%)	
		Intervention	Control
Demographics			
Sex			
Male	840 (48.9)	415/856 (48.5)	425/862 (49.3)
Female	878 (51.1)	441/856 (51.5)	437/862 (50.7)
Age group			
<5 y	1302 (75.8)	653/856 (76.3)	649/862 (75.3)
5 to <10 y	305 (17.7)	146/856 (17.0)	159/862 (18.4)
10 to <18 y	111 (6.5)	57/856 (6.7)	54/862 (6.3)
Reason for consultation			
Fever	1668 (97.1)	834 (97.4)	834 (96.8)
Redness of the eyes	15 (0.9)	9 (1.1)	6 (0.7)
Eye discharge	34 (2.0)	26 (3.0)	8 (0.9)
Sore throat	18 (1.0)	15 (1.8)	3 (0.3)
Ear discharge	15 (0.9)	9 (1.1)	6 (0.7)
Swelling behind the ear	1 (0.1)	1 (0.1)	0 (0.0)
Sneezing and rhinorrhea	739 (43.0)	433 (50.6)	306 (35.5)
Postnasal drip	0 (0)	0 (0.0)	0 (0.0)
Cough	671 (39.1%)	345 (40.3)	326 (37.8)
Chest pain	6 (0.3)	5 (0.6)	1 (0.1)
Diarrhea	373 (21.7)	187 (21.8)	186 (21.6)
Vomiting	443 (25.8)	212 (24.8)	231 (26.8)
Pain while swallowing	22 (1.3)	12 (1.4)	10 (1.2)
Abdominal pain	392 (22.8)	226 (26.4)	166 (19.3)
Dysuria	10 (0.6)	6 (0.7)	4 (0.5)
Urinary frequency or urgency	1 (0.1)	1 (0.1)	0 (0.0)
Rash	61 (3.6)	26 (3.0)	35 (4.1)
Headache	250 (14.6)	130 (15.2)	120 (13.9)
Neck stiffness	0 (0)	0 (0.0)	0 (0.0)
Photophobia	0 (0)	0 (0.0)	0 (0.0)
Joint pain or swelling	0 (0)	0 (0.0)	0 (0.0)
Other	66 (3.8)	24 (2.8)	42 (4.9)
Suspicion of respiratory tract infection			
Yes	904 (52.6)	398 (46.2)	506 (59.2)
No	813 (47.4)	464 (53.8)	349 (40.8)
Diagnostic test performed			
CRP (mg/L)	…	788 (92.1)	…
<20	…	338 (42.9)	…
20 to <80	…	245 (31.1)	…
≥80	…	205 (26.0)	…
WBC counts (counted/microliter)	…	787 (91.9)	…
<11 000	…	717 (91.8)	…
≥11 000	…	70 (8.9)	…
Urine dipstick	…	5 (0.6)	…
Malaria RDT	1718 (100.0)	856 (100.0)	862 (100.0)
Typhoid RDT	…	279 (32.6)	…
*Streptococcus* A RDT	…	36 (4.2)	…
*Streptococcus pneumoniae* RDT (for ≥5 y)	…	24 (2.8)	…
Influenzae A/B RDT	…	379 (44.3)	…
RSV RDT (for <5 y)	…	136 (15.9)	…
Dengue NS1/IgM RDT	…	53 (6.2)	…

Abbreviations: IgM, immunoglobulin M; RDT, rapid diagnostic test; RSV, respiratory syncytial virus; WBC, white blood cell.

Prior to testing, a presumptive diagnosis of malaria was reported in 31.9% of cases. Post-testing, 75% of cases were confirmed as malaria. All patients received a malaria RDT (100%; 1718/1718) as per standard practice at the recruitment health centers. CRP (92.1%; 788/856) and WBC (91.9%; 787/856) were systematically tested in the intervention arm (results shown in Supplementary Table 1). Pathogen-specific POCTs were performed less frequently, ranging from influenza A/B RDT (44.3%; 379/856) to urine dipstick (0.6%; 5/856 of participants). Baseline characteristics are presented in Table 1.

For the 788 CRP test results, 338 mg/L (42.9%), 245 mg/L (31.1%), and 205 mg/L (26.0%) were in the categories of less than 20, 20–80, and greater than 80, respectively. For WBC total counts, 529 counted/microliter (67.2%) and 258 counted/microliter (32.8%) were less than 11 000 and 11 000 or more, respectively. For neutrophil counts, 717 (91.1%) and 70 (8.9%) were <75% and ≥75%, respectively.

### Outcomes

No significant difference was seen between the 2 arms in the proportion of favorable clinical outcomes at the day 7 follow-up visit: 99.5% (840/844) in the intervention arm versus 100% (837/837) in the control arm (RD: −0.5; 95% CI: −1.1 to .1; *P* = .135). This was consistent across age groups, sites, and seasons (Table 2).

**Table 2.** Clinically Favorable Outcome Stratified by Age Group and Yearly Quarter/Season in the Randomized Group

Characteristics	Overall		Intervention Arm		Control Arm		Risk Difference,% [95% CI]	*P*
	n/N (%)	95% CI	n/N (%)	95% CI	n/N (%)	95% CI		
All	1677/1681 (99.8)	99.4–99.9	840/844 (99.5)	98.8–99.8	837/837 (100.0)	99.5–100.0	−0.5 [−1.1 to .1]	.135
Age group								
<5 y	1268/1271 (99.8)	99.3–99.9	639/642 (99.5)	98.6–99.8	629/629 (100.0)	99.4–100	−0.5 [−1.2 to .2]	.255
5 to <18 y	409/410 (99.8)	98.6–100.0	201/202 (99.5)	97.2–99.9	208/208 (100.0)	98.2–100.0	−0.5 [−2.0 to 1.0]	.988
5 to <10 y	300/301 (99.7)	98.1–99.9	144/145 (99.3)	96.2–99.9	156/156 (100.0)	97.6–100.0	−0.7 [−2.7 to 1.3]	.971
10 to <18 y	109/109 (100.0)	96.6–100.0	57/57 (100.0)	93.7–100.0	52/52 (100.0)	93.1–100.0	0.0 [0.0 to 0.0]	NA
Yearly quarter/season								
September	178/182 (97.8)	94.5–99.1	88/92 (95.7)	89.3–98.3	90/90 (100.0)	95.9–100.0	−4.3 [−9.6 to .9]	.135
October–December	512/512 (100.0)	99.3–100.0	257/257 (100.0)	98.5–100.0	255/255 (100.0)	98.5–100.0	0.0 [0.0 to 0.0]	NA
January–March	404/404 (100.0)	99.1–100.0	208/208 (100.0)	98.2–100.0	196/196 (100.0)	98.1–100.0	0.0 [0.0 to 0.0]	NA
April–June	233/233 (100.0)	98.4–100.0	109/109 (100.0)	96.6–100.0	124/124 (100.0)	97.0–100.0	0.0 [0.0 to 0.0]	NA
July–September	350/350 (100.0)	98.9–100.0	178/178 (100.0)	97.9–100.0	172/172 (100.0)	97.8–100.0	0.0 [0.0 to 0.0]	NA
Dry season	729/729 (100.0)	99.5–100.0	358/358 (100.0)	98.9–100.0	371/371 (100.0)	99.0–100.0	0.0 [0.0 to 0.0]	NA
Rainy season	948/952 (99.6)	98.9–99.8	482/486 (99.2)	97.9 –99.7	466/466 (100.0)	99.2–100.0	−0.8 [−1.8 to .2]	.144
Sub-stratification by age and season								
<5 y								
Dry season	554/554 (100.0)	99.3–100.0	279/279 (100.0)	98.6–100.0	275/275 (100.0)	98.6–100.0	0.0 [0.0 to 0.0]	NA
Rainy season	714/717 (99.6)	98.8–99.9	360/363 (99.2)	97.6–99.7	354/354 (100.0)	98.9–100.0	−0.8 [−2.0 to .4]	.256
5 to <18 y								
Dry season	175/175 (100.0)	97.9–100.0	79/79 (100.0)	95.4–100.0	96/96 (100.0)	96.2–100.0	0.0 [0.0 to 0.0]	NA
Rainy season	234/235 (99.6)	97.6–99.9	122/123 (99.2)	95.5–99.9	112/112 (100.0)	96.7–100.0	−0.8 [−3.2 to 1.6]	1.000
Diagnosis in nonrespiratory group								
Malaria positive	1253/1257 (99.7)	99.2–99.9	634/638 (99.4)	98.4–99.8	619/619 (100.0)	99.4–100.0	−0.6 [−1.4 to .1]	.141
Malaria negative	424/424 (100.0)	99.1–100.0	206/206 (100.0)	98.2–100.0	218/218 (100.0)	98.3–100.0	0.0 [0.0 to 0.0]	NA
Primary presumptive diagnosis								
Respiratory diagnosis	829/829 (100.0)	99.5–100.0	460/460 (100.0)	99.2–100.0	369/369 (100.0)	99.0–100.0	0.0 [0.0 to 0.0]	NA
Nonrespiratory diagnosis	848/852 (99.5)	98.8–99.8	380/384 (99.0)	97.4–99.6	468/468 (100.0)	99.2–100.0	−1.0 [−2.3 to .2]	.087
CRP								
<20	…	…	332/333 (99.7)	98.3–100.0	…	…	…	
20 to <80	…	…	242/243 (99.6)	97.7–99.9	…	…	…	
≥80	…	…	199/201 (99.0)	96.5–99.7	…	…	…	
WBC counts								
<11 000	…	…	522/524 (99.6)	98.6–99.9	…	…	…	
≥11 000	…	…	250/252 (99.2)	97.2–99.8	…	…	…	
Neutrophils								
<75%	…	…	702/706 (99.4)	98.6–99.8	…	…	…	
≥75%	…	…	70/70 (100.0)	94.8–100.0	…	…	…	

Abbreviations: CI, confidence interval; CRP, C-reactive protein; NA, not applicable; WBC, white blood cell.

The antibiotic prescription rates were significantly lower in the intervention arm (Table 3): 40.6% (343/844) versus 57.5% (481/837) in the control arm (RR: 29.3%; 95% CI: 21.8–36.0%; *P* < .001; RD: −16.8%: 95% CI: −21.7% to −12.0%; *P* < .001). This translates into a number needed to test of 6 (95% CI: 5–8), meaning that, when applied, the intervention resulted in 1 fewer antibiotic prescription every 6 consultations.

**Table 3.** Antibiotic Prescription Stratified by Age Group and Yearly Quarter/Season in the Randomized Group

Characteristics	Overall		Intervention Arm		Control Arm		Risk Difference, % [95% CI]	Relative Risk, % [95% CI]	*P*
	n/N (%)	95% CI	n/N (%)	95% CI	n/N (%)	95% CI			
All	824/1681 (49.0)	46.6–51.4	343/844 (40.6)	37.4–44.0	481/837 (57.5)	54.1–60.8	−16.8 [−21.7 to −12.0]	−29.3 [−21.8 to −36.0]	**<**.**001**
Age group									
<5 y	667/1271 (52.5)	49.7–55.2	272/642 (42.4)	38.6–46.2	395/629 (62.8)	59.0–66.5	−20.4 [−26.0 to −14.9]	−32.5 [−24.8 to −39.5]	**<**.**001**
>5 y	157/410 (38.3)	33.7–43.1	71/202 (35.1)	28.9–41.9	86/208 (41.3)	34.9–48.1	−6.2 [−16.1 to 3.7]	−15.0 [−33.6 to 8.9]	.234
5 to <10 y	114/301 (37.9)	32.6–43.5	57/145 (39.3)	31.7–47.4	57/156 (36.5)	29.4–44.3	2.8 [−8.9 to 14.4]	7.6 [−19.4 to 43.7]	.707
10 to <18 y	43/109 (39.4)	30.8–48.8	14/57 (24.6)	15.2–37.1	29/52 (55.8)	42.3–68.4	−31.2 [−50.6 to −11.8]	−56.0 [−73.7 to −26.3]	.**002**
Yearly quarter/season									
September 2020	53/182 (29.1)	23.–36.1	19/92 (20.7)	13.6–30.0	34/90 (37.8)	28.5–48.1	−17.1 [−31.2 to −3.0]	−45.3 [−66.2 to −11.6]	.**017**
October–December 2020	257/512 (50.2)	45.9–54.5	103/257 (40.1)	34.3–46.2	(154/255 (60.4)	54.3–66.2	−20.3 [−29.2 to −11.4]	−33.5 [−44.5 to −20.6]	**<**.**001**
January–March 2021	247/404 (61.1)	56.3–65.8	110/208 (52.9)	46.1–59.6	137/196 (69.9)	63.1–75.9	−17.0 [−26.8 to −7.2]	−24.3 [−35.4 to −11.4]	**<**.**001**
April–June 2021	156/233 (67.0)	60.7–72.7	50/109 (45.9)	36.8–55.2	106/124 (85.5)	78.2–90.6	−39.6 [−51.7 to −27.5]	−46.3 [−56.8 to −33.4]	**<**.**001**
July–September 2021	111/350 (31.7)	27.1–36.8	(61/178 (34.3)	27.7–41.5	50/172 (29.1)	22.8–36.2	5.2 [−5.1 to 15.5]	17.9 [−13.5 to 60.7]	.352
Dry season	453/729 (62.1)	58.6–65.6	179/358 (50.0)	44.9–55.1	274/371 (73.9)	69.2–78.1	−23.9 [−31.0 to −16.7]	−32.3 [−40.0 to −23.7]	**<**.**001**
Rainy season	(371/952 (39.0)	35.9–42.1	164/486 (33.7)	29.7–38.1	207/466 (44.4)	40.0–49.0	−10.7 [−17.1 to −4.3]	−24.0 [−35.3 to −10.8]	**<**.**001**
Sub-stratification by age and season									
<5 y									
Dry season	367/554 (66.2)	62.2–70.1	146/279 (52.3)	46.5–58.1	221/275 (80.4)	75.3–84.6	−28.0 [−35.9 to −20.2]	−34.9 [−42.6 to −26.1]	**<**.**001**
Rainy season	300/717 (41.8)	38.3–45.5	126/363 (34.7)	30.0–39.7	174/354 (49.2)	44.0–54.3	−14.4 [−21.9 to −7.0]	−29.4 [−40.8 to −15.8]	**<**.**001**
5 to <18 y									
Dry season	86/175 (49.1)	41.8–56.5	33/79 (41.8)	31.5–52.8	53/96 (55.2)	45.2–64.8	−13.4 [−29.3 to 2.5]	−24.3 [−44.9 to 3.8]	.106
Rainy season	71/235 (30.2)	24.7–36.4	38/123 (30.9)	23.4–39.5	33/112 (29.5)	21.8–38.5	1.4 [−11.2 to 14.0]	4.9 [−29.0 to 54.8	.923
Diagnosis in nonrespiratory group									
Malaria positive	559/1257 (44.5)	41.7–47.2	263/638 (41.2)	37.5–45.1	296/619 (47.8)	43.9–51.8	−6.6 [−12.2 to 1.0]	−13.8 [−23.4 to −2.4]	.**022**
Malaria negative	265/424 (62.5)	57.8–67.0	80/206 (38.8)	32.4–45.6	185/218 (84.9)	79.5–89.0	−46.0 [−54.7 to −37.4]	−54.2 [−61.8 to −45.2]	**<**.**001**
Primary presumptive diagnosis									
Respiratory diagnosis	586/829 (70.7)	67.5–73.7	247/460 (53.7)	49.1–58.2	339/369 (91.9)	88.6–94.2	−38.2 [−43.8 to −32.6]	−41.6 [−46.6 to −36.0]	**<**.**001**
Nonrespiratory diagnosis	238/852 (27.9%)	25.0–31.0	96/384 (25.0)	20.9–29.6	142/468 (30.3)	26.4–34.6	−5.3 [−11.6 to 0.9]	−17.6 [−33.9 to 2.8]	.098
CRP									
<20	…	…	87/333 (26.1)	21.7–31.1	…	…	**…**	…	
20 to <80	…	…	73/243 (30.0)	24.6–36.1	…	…	**…**	…	
≥80	…	…	117/201 (58.2)	51.3–64.8	…	…	**…**	…	
WBC counts									
<11 000	…	…	136/524 (26.0)	22.4–29.9	…	…	**…**	…	
≥11 000	…	…	141/252 (56.0)	49.8–62.0	…	…	**…**	…	
Neutrophils									
<75%	…	…	232/706 (32.9)	29.5–36.4	…	…	…	…	
≥75%	…	…	45/70 (64.3)	52.6–74.5	…	…	…	…	

Abbreviations: CI, confidence interval; CRP, C-reactive protein; WBC, white blood cell.

The bold *P* represent the statistical significant values.

Antibiotic prescriptions were significantly reduced in patients who tested negative for malaria (RR: 54.2%; 95% CI: 45.2–61.8%; RD: −46.0%; 95% CI: −54.7% to −37.4%; *P* < .001) and patients who tested positive for malaria (RR: 13.8%; 95% CI: 2.4–23.8%; RD: −6.6%; 95% CI: −12.2% to −1.0%; *P* = .022), and those with a respiratory diagnosis (RR: 41.6%; 95% CI: 36.0–46.6%; RD: −38.2%; 95% CI: −43.8% to −32.6%; *P* < .001). The intervention did not result in a reduction in antibiotic prescriptions in patients with a nonrespiratory diagnosis (RD: −5.3%; 95% CI: −11.6% to 0.9%; *P* = .098) or in those with a diagnosis of pneumonia (7.5% [64/856] in the intervention arm vs 9.8% [83/862] in the control arm).

The reduction in antibiotic prescriptions was statistically significant in children aged 6–59 months (RD: −20.4%; 95% CI: −26.0% to −14.9%; *P* < .001) and 10- to less-than-18-year-olds (RD: −31.2%; 95% CI: −50.6% to −11.8%; *P* = .002), but not in children aged 5 to less than 10 years old (RD: −2.8%; 95% CI: −8.9% to 14.4%; *P* = .707). When stratified by seasonality, the reduction was significant in both the dry and rainy seasons, and also in 3 of the year quarters, except for July–September 2021 (Table 3). When sub-stratified by age group and seasonality, the reduction was statistically significant in the dry season (RD: −28.0%; 95% CI: −35.9% to −20.2%; *P* < .001) and the rainy season (RD: −14.4%; −21.9% to −7.0%; *P* < .001) in young children aged 6–59 months. However, for participants aged 5 to less than 18 years, the analysis shows a reduction in antibiotic prescriptions during the dry season only, although this reduction was not statistically significant (RD: −13.4%; 95% CI: −29.3% to 2.5%; *P* = .106; Table 3).

In the intervention arm, antibiotic prescription rates, but not adverse clinical outcomes, increased with CRP level and WBC and neutrophil counts (Supplementary Table 1).

### Secondary Objective 1: Adherence to the Prescriptions by Patients

Data on adherence to antibiotic prescriptions are reported in Table 4. Adherence to an antibiotic prescription, or not taking an antibiotic if not prescribed, was statistically higher in the intervention arm: 91.3% (756/828) *versus* 87.7% (711/811) in the control arm (*P* = .020), measured through qualitative interviews, direct report, and pill count. Adherence to antibiotic prescriptions alone did not differ between the 2 arms (*P* > .05)

**Table 4.** Adherence to Antibiotic Prescription at Day 0 in Patients Managed in the Intervention and Control Arms

Characteristics	Overall, n/N (%)		Intervention Arm, n/N (%)		Control Arm, n/N (%)		Risk Difference, % [95% CI]	*P*
	Yes	No	Yes	No	Yes	No		
Antibiotic prescription	823/1678 (49.0)	855/1678 (51.0)	343/842 (40.7)	499/842 (59.3)	480/836 (57.4)	356/836 (42.6)	−16.7 [−21.5 to −11.8]	**<**.**001**
Primary adherence measurement (C + D)	1467/1639 (89.5)	172/1639 (10.5)	756/828 (91.3)	72/828 (8.7)	711/811 (87.7.3)	100/811 (12.3)	3.6 [0.5 to 6.7]	.**020**
(Secondary measurement) Adherence A	788/823 (95.7)	35/823 (4.3)	331/343 (96.5)	12/343 (3.5)	457/480 (95.2)	23/480 (4.8)	1.3 [−1.7 to 4.3]	.465
(Secondary measurement) Adherence B	615/778 (79.0)	163/778 (21.0)	257/328 (78.4)	71/328 (21.6)	358/450 (79.6)	92/450 (20.4)	−1.2 [−7.3 to 4.9]	.751
(Secondary measurement) Adherence C	613/784 (78.2)	171/784 (21.8)	257/329 (78.1)	72/329 (21.9)	356/455 (78.2)	99/455 (21.8)	−0.1 [−6.1 to 5.9]	1.000
(Secondary measurement) Adherence D	854/855 (99.9)	1/855 (0.1)	355/356 (99.7)	1/356 (0.3)	499/499 (100.0)	0/499 (0)	0.3 [−0.5 to 1.1]	.865
(Secondary measurement) Adherence A + D	1642/1678 (97.9)	36/1678 (2.1)	830/842 (98.6)	12/842 (1.4)	812/836 (97.1)	24/836 (2.9)	1.4 [−0.06 to 3.0]	.061
(Secondary measurement) Adherence B + D	1469/1633 (90.0)	164/1633 (10.0)	756/827 (91.4)	71/827 (8.6)	713/806 (88.5)	93/806 (11.5)	3.0 [−0.09 to 6.0]	.057

Abbreviation: CI, confidence interval.

Question: “patient bought antibiotic” and question: “other antibiotic” were common criteria to (A), (B) and (C)—(A) accounting for the following question: “Completed treatment as reported through qualitative in-depth-interviews, or directly to the healthcare worker”; (B) accounting for the following question: “Pill count criterion (≥90%)”; and (C) accounting for the following questions: “Completed treatment” and “Pill count criterion (≥90%)”. The nonadherent cases directly reflect cases being referred to as “antibiotic taken”—(D) accounting for the following question: “No other antibiotic” [in subjects without antibiotic prescription on day 0].”

The bold *P* represent the statistical significant values.

## DISCUSSION

This study shows the potential of interventions to improve the diagnosis and management of febrile diseases and reduce inappropriate prescriptions of antibiotics in children and young people under 18 years attending primary outpatient facilities without compromising clinical outcomes in Burkina Faso. The intervention resulted in significant reductions in antibiotic prescriptions overall, both in relative (29%) and absolute terms (17%), which would save 1 inappropriate antibiotic prescription for every 6 children and adolescents consulting for fever at our peripheral healthcare facilities. The effect was seen in children under 5 years and in those between 10 and 18 years of age.

This approach also lends itself to further targeting the intervention, as the largest gains were obtained in patients who tested negative for malaria (1 fewer prescription for every 5 tested) and those with a respiratory presentation (1 fewer prescription for every 6 tested).

These effects in malaria-negative cases are particularly important, since an unfortunate side-effect of the WHO's recommendation to confirm malaria infection through rapid diagnostic testing prior to the administration of antimalarial treatment [19] has been an increase in antibiotic prescriptions in patients with a negative malaria test [15]. Although 47.8% of patients with malaria also received an antibiotic in routine practice, this was significantly less in the intervention arm (41.2%) and was statistically significant (difference = 6.6%; *P* = .022). While this could be a precautionary measure when multiple infections cannot be excluded, better training and awareness by healthcare workers should further decrease this practice.

In the control arm, the antibiotic-prescribing rate with routine practice was approximately 57.5% which is 20% lower than expected based on previous years in these facilities [20]. Indeed, children under 5 years enrolled in this study represent approximately 76% of the sample size and those randomized in the control arm are managed with IeDA. It is obvious that this difference reported in the control arm (post-IeDA era) compared with previous years (pre-IeDA era) may be ascribed to the routine implementation of IeDA in children under 5 years (3 in 4 attending the outpatient clinic) in primary health facilities for the management of outpatient illnesses [23]. However, despite the implementation of IeDA as part of standard practice for the management of diseases in children under 5 years in the routine-care system, antibiotics were almost systematically prescribed to patients with nonmalaria infection and respiratory diagnosis in the control arm (84.9% and 91.9%) compared with the intervention arm (38.8% and 53.7%).

A more substantial reduction in antibiotic prescription was observed in the dry season (28%) compared with the rainy season (14.4%). Several factors may explain these findings: (1) the limits of histidine-rich protein 2 (HRP2)-based RDTs could hide a potential bacterial infection in the intervention arm [15,24]; (2) the fear of overlooking a potential and treatable bacterial infection in the control arm could lead to antibiotic misuse; and (3) CRP levels can be elevated with a malaria infection, which could also lead to antibiotic misuse [25–27], especially as a previous study reported a very small proportion of coinfections that required antibiotic treatment [3]. To make the intervention more accurate while not missing a potential and treatable bacterial infection with malaria false-positive tests [24], there is a clear medical need for more accurate diagnostic tools/approaches in endemic areas and practical tools specific to bacterial infections only.

The diagnostic algorithm used in this study recommended that CRP and WBC be tested systematically on all patients in the intervention arm, while the use and choice of pathogen-specific POCTs should be based on clinical presentation according to age. Exceptions to this were children under 5 years who met the criteria for pneumonia (as defined by the WHO) or otitis. In this study, approximately 10.4% (otitis: 2.7%; pneumonia: 7.5%) of children aged 6–59 months in the intervention arm met the clinical criteria for antibiotic prescription based on clinical outcomes alone versus 9.6% in standard practice. Such a small rate of antibiotic prescriptions reported in both arms was based on clinical examination only. This low level of antibiotic prescriptions has also been reported in previous studies of IeDA [23,28,29].

Last, the results of this study provide evidence that the intervention in patients aged 5–10 years may be associated with increased antibiotic prescriptions compared with standard of care. However, all cases of hospitalization and treatment failures in this age group were reported in standard practice. This may be because patients in this age group attending outpatient facilities were nonsevere cases, most of whom who did not need antibiotics to improve their clinical outcome. As mentioned previously, the limitations of HRP2-based RDTs and CRP tests to correctly identify patients who require antibiotics could also explain these findings.

The improved adherence to prescriptions reported in this study as a result of the implementation of biological POCTs is in line with other studies conducted in LMICs [21,30,31]. Indeed, participants in the intervention arm were more likely to follow healthcare prescriptions, suggesting that, in outpatient facilities, the lack of a T&C package may have contributed to lower adherence to antibiotic prescriptions. The reasonable waiting time of participants in intervention arm can also be a factor that also influenced the adherence to healthcare prescriptions.

The study has some limitations. First, this was an individual randomized controlled trial where the intervention package, including the T&C package, was designed for participants in the intervention arm only. The impact of the intervention might have been underestimated as a consequence of potential contamination of arms—that is, that the impact of the T&C package could be diluted due to participants and/or caregivers in both the intervention and control arms living in the same areas. Second, as this was a diagnostic trial conducted at an outpatient clinic and health facility, there is the possibility that nurses' adherence to clinical criteria may have led to deviations from the predefined protocol.

In conclusion, the proposed intervention package has the potential to reduce unnecessary prescription of antibiotics compared with standard practice without affecting clinical outcomes. The size of the effect varied with age and season, and was particularly marked in patients who tested negative for malaria and those with a respiratory diagnosis.

## Supplementary Data

Supplementary materials are available at *Clinical Infectious Diseases* online. Consisting of data provided by the authors to benefit the reader, the posted materials are not copyedited and are the sole responsibility of the authors, so questions or comments should be addressed to the corresponding author.

## Supplementary Material

ciad331_Supplementary_Data

## Data Availability

For any data request, please contact the Project Manager at juvenal.nkeramahame@finddx.org and the local Principal Investigator Halidou Tinto (by e-mail at halidoutinto@gmail.com).

## Notes

***Author Contributions.*** P. O., O. S., H. T., and F. K. conceived and designed the study; F. K., D. V., B. K., A. N. K., T. R., O. S., S. S., A. C., and H. T. supervised the implementation of the study in the field and data collection; F. K., D. V., B. K., T. R., H. T., and J. N. analyzed and drafted the paper; all the coauthors revised the manuscript; H. T., P. O., and J. N. supervised the writing of the manuscript.

***Acknowledgments.*** The authors thank all the participants and their parents or guardians for their participation. They acknowledge the National Ethical Committee for Research in Health Science and the Institutional Ethical Committee of the Institut de Recherche en Sciences de la Santé (IRSS). They acknowledge the staff of IRSS-CRUN to provide technical support during the study implementation. They also extend their thanks to the Health District of Nanoro, the staff of the Medical Center Saint Louis of Temnaore, and the health facility of Pella.

***Financial support*.** This work was supported by the Swiss Agency for Development and Cooperation (SDC), the Federal Ministry of Economic Cooperation and Development (BMZ), and the UK Department for International Development (DFID) (now the Foreign, Commonwealth and Development Office [FCDO]).

***Supplement sponsorship.*** This article appears as part of the supplement “Using Diagnostic Tools to Support Antimicrobial Stewardship and Improve Outcome in Resource-Limited Contexts,” sponsored by FIND.
