# Supplementary material for: A Randomized Trial to Assess the Impact of a Package of Diagnostic Tools and Diagnostic Algorithm on Antibiotic Prescriptions for the Management of Febrile Illnesses Among Children and Adolescents in Primary Health Facilities in Burkina Faso
Source: Clin Infect Dis. 2023 Jul 25;77(Suppl 2):S134–44. doi: 10.1093/cid/ciad331 (PMC10368409; doi:10.1093/cid/ciad331)
Supplement: ciad331_Supplementary_Data [file ciad331_supplementary_data.docx]

## Supplementary Data

#### Supplementary Table 1.

|  | **Diagnostic test result** | | **Favorable clinical outcome** | | **Antibiotic prescriptions** | | |
| --- | --- | --- | --- | --- | --- | --- | --- |
|  | n/N (%) | 95% CI | n/N (%) | 95% CI | n/N (%) | 95% CI |  |
| C-reactive protein | 776/856 (90.6) |  | 332/333 (99.7) | 98.3–100.0 | 87/333 (26.1) | 21.7–31.1 |  |
| <20 | 333 (42.9) |  | 242/243 (99.6) | 97.7–99.9 | 73/243 (30.0) | 24.6–36.1 |  |
| 20 to <80 | 243 (31.3) |  | 199/201 (99.0) | 96.5–99.7 | 117/201 (58.2) | 51.3–64.8 |  |
| ≥80 | 201 (25.9) |  |  |  |  |  |  |
| White blood cell counts |  |  | 522/524 (99.6) | 98.6–99.9 | 136/524 (26.0) | 22.4–29.9 |  |
| <11,000 | 524 (67.5) |  | 250/252 (99.2) | 97.2–99.8 | 141/252 (56.0) | 49.8–62.0 |  |
| ≥11,000 | 252 (32.5) |  |  |  |  |  |  |
| Neutrophils |  |  | 702/706 (99.4) | 98.6–99.8 | 232/706 (32.9) | 29.5–36.4 |  |
| <75% | 706 (91) |  | 70/70 (100.0) | 94.8–100.0 | 45/70 (64.3) | 52.6–74.5 |  |
| ≥75% | 70 (9) |  |  |  |  |  |  |

CI, confidence interval.
